# Relationship between alexithymia and quality of life in intensive care worker: a correlational study

**DOI:** 10.1097/MS9.0000000000005024

**Published:** 2026-04-27

**Authors:** Areeba Anam, Moazzma Yahya, Bisma Bashir Ahmad, Bakhtawar Aqeel, Ishwa Khan, Warda Fatima Shafee, Sumia Fatima, Muddassir Khalid

**Affiliations:** a Department of Medicine, Allama Iqbal Medical College, Lahore, Pakistan; b Department of Medicine, Lahore General Hospital, Lahore, Pakistan; c Department of Medicine, People’s University of Medical and Health Sciences for Women, Nawabshah, Pakistan; d Department of Medicine, Fazaia Medical College, Islamabad, Pakistan; e Department of Medicine, Nishtar Medical University, Multan, Pakistan; f Department of Medicine, Rawalpindi Medical University, Rawalpindi, Pakistan

**Keywords:** behavior, emotions, intensive care units, mood disorders: affective symptoms, nurses, prevalence

## Abstract

**Objectives:**

The purpose of this study is to establish the prevalence of alexithymia among intensive care unit doctors and nurses, as well as to investigate the relationship between alexithymia and quality of life. It also looks at how demographic variables, including age, gender, occupation, career, health difficulties, marital status, and quality of life, affect the occurrence of alexithymia.

**Methods:**

The study used a cross-sectional, multicenter design with intensive care unit (ICU) healthcare workers from multiple centers. To determine the existence of alexithymia, participants completed the 20-item Toronto Alexithymia Scale (TAS-20) questionnaire. To assess quality of life, the World Health Organization’s abbreviated version questionnaire, which contains 26 items, was used. The full questionnaire’s 100 items were not used in this study. Two of the 26 elements of the abbreviated version were generic in character, while the other 24 things were divided into four domains.

**Results:**

According to the study, physicians exhibited a higher prevalence of alexithymia compared to nurses; 22% of the physicians were diagnosed with alexithymia, while it was 0% in nurses. No significant associations were found between alexithymia and gender (*P* = 0.198), age (*P* = 0.347), or marital status (*P* = 0.103). Although Alexithymia showed statistically significant associations with certain reported health issues (seasonal illness, GIT disorders, stress, anxiety, headache, and psychological issues), it was overall more frequent in individuals who perceived themselves as generally healthy (18%) than in those who reported being ill (2%). Alexithymia showed significantly negative correlation with all the four subscales of quality of life (i.e., physical health, psychological health, social relations, and environmental quality). However, no significant association was observed with single item overall quality-of-life measure. Furthermore, there was no significant correlation between quality of life and the three subscales of alexithymia (i.e., identifying feelings, describing feelings, and externally oriented thinking).

**Conclusion:**

The results of this study demonstrate a notable prevalence of alexithymia among ICU healthcare workers, with a higher frequency among physicians compared to nurses. Alexithymia was not significantly associated with marital status. More individuals with alexithymia reported being generally healthy, suggesting that the absence of physical illness does not rule out emotional processing difficulties. Although alexithymia was not significantly correlated with overall quality of life, it showed significant negative associations with four domain-specific aspects. These findings suggest that emotional processing difficulties may be associated with specific dimensions of well-being among ICU healthcare workers, highlighting the need for further research on emotional awareness, quality of life, psychological well-being, and occupational stress in healthcare settings.

## Introduction

“Alexithymia” is a term derived from Greek roots that literally means “no words for emotions” or “lack of words to express emotions.” It is used to describe a condition where an individual has difficulty identifying and expressing their feelings^[^1,2^]^. The Toronto Alexithymia Scale (TAS-20) divides alexithymia into three main subtypes: difficulty identifying emotions, difficulty describing emotions, and externally orientated feelings^[^3,4^]^.

In healthy individuals, there is generally a strong correlation between implicit indicators of emotional reactivity like physiological arousal and explicit indicators, such as the intensity of self-reported emotions. In contrast, those with alexithymia experience a disconnect between these implicit and explicit emotional responses^[^4–6^]^. High levels of alexithymia can affect a person’s social life and also cause stress. People with alexithymia often experience higher levels of anxiety, depression, and psychological distress. They may also report more physical symptoms that have no medical explanation and suffer from emotional instability due to their limited psychological resources. Additionally, individuals with alexithymic traits may be at an increased risk of having suicidal thoughts^[^7^]^.

Various studies indicate that the prevalence of alexithymia in the general population ranges from 9 to 23%^[^7–10^]^. A research study in China reported the occurrence of alexithymia in intensive care unit (ICU) nurses to be 43.7%^[^1^]^. A study by Khan^[^11^]^ on the prevalence of alexithymia among Pakistani adults found that the condition is present in the society, with men (93%) being more susceptible than women (87%)^[^12^]^.

Quality of life (QOL) seeks to measure the well-being of a population, considering both the positive and negative aspects of their life. The World Health Organization (WHO) defines QOL as an individual’s perception of their life in relation to their personal goals and cultural context. The University of Toronto explains QOL as the possibilities of how much an individual can enjoy the opportunities in their life^[^13^]^.

There are several studies that have investigated the effect of alexithymia on a person’s QOL^[^14^]^. Researchers have examined the relationship between alexithymia and perceived QOL in patients with various diseases, such as Parkinson’s disease, Hashimoto’s thyroiditis, and irritable bowel syndrome^[^15–17^]^.

However, there is limited research on the correlation between alexithymia and QOL among healthcare workers. Our research aims to examine the prevalence of alexithymia among critical care unit (CCU) workers and to explore its association with their perceived QOL. Understanding the relationship between emotional processing and perceived QOL in healthcare workers is important, as occupational stress and emotional suppression are common in intensive care settings.

This cross-sectional study has been reported in line with the STROCSS guidelines^[^18^]^.

## Methodology

A quantitative cross-sectional study was conducted in accordance with (1) the Strengthening the Reporting of Observational Studies in Epidemiology (STROBE)^[^19^]^ and (2) the Consensus-Based Checklist for Reporting of Survey Studies (CROSS)^[^20^]^ guidelines. A convenient non-probability sampling strategy was employed due to logistical constraints. This research was done in ICUs in Rawalpindi.

We calculated the required sample size using OpenEpi software (Version 3.01), assuming a confidence level of 95% and a margin of error of 5%. There were about 150 CCU workers in the hospital. The calculation yielded a minimum sample size of 100. This study was conducted on doctors and nurses working in ICUs for a total period of 2 months.

A total of three assessment measures were used in this study, i.e., the personal assessment sheet, the TAS-20, and the World Health Organization QOL Brief Version (WHOQOL-BREF).

A 5-point Likert scale was used to rate the responses (1 being strongly disagree and 5 being strongly agree). Higher total scores for the five reverse-scored items (4, 5, 10, 18, and 19) indicate higher levels of alexithymia (≤51: no alexithymia; 52–60: potential cases of alexithymia; and ≥61: alexithymia).

The WHOQOL-BREF, a 26-item survey on a 5-point Likert scale, was used to evaluate QOL. QOL was primarily analyzed as a continuous variable across WHOQOL-BREF domains, consistent with WHO recommendations. For descriptive purposes only, scores ≥60 were considered as relatively better perceived QOL based on thresholds reported in previous regional studies; however, inferential analyses were conducted using continuous scores^[^21–23^]^.

HIGHLIGHTS

- Alexithymia was prevalent among intensive care unit staff, with higher rates observed in physicians.
- No significant association was found between alexithymia and age, gender, or marital status.
- Alexithymia correlated with multiple self-reported health and psychological conditions.
- Significant links existed between alexithymia and all quality-of-life subdomains.
- Overall quality of life was not significantly affected by the presence of alexithymia.

### Statistical analysis

The data of this study were analyzed using SPSS version-27. To find out the reliability of the scales used in this study, a reliability analysis was used (as reported in Table 1). Descriptive statistics were used to summarize demographic characteristics. Associations between categorical variables were examined using chi-square tests of independence, i.e., to examine the relationship of alexithymia with different categories of demographics, as shown in Table 3. The TAS-20 and WHOQOL-BREF scores were calculated as composite scores from multiple Likert-scale items and were therefore treated as approximately continuous variables. Pearson correlation coefficients were used to examine associations between alexithymia scores and QOL domains (see Table 4). A *P*-value of <0.05 was considered statistically significant.

**Table 1:** Descriptive statistics and reliability of the study variables.

				**Range**		
**Variables**	***k***	**M**	**SD**	**Potential**	**Actual**	***α***
Alexithymia	20	52.80	11.16	20–100	29–84	.83
Quality of life	26	89.67	16.10	–	–	.92
Physical health		13.89	2.71		8–19.43	
Psychological health		13.43	2.82		7.33–18.67	
Social relations		13.07	3.68		4–20	
Quality of environment		13.92	2.97		8–19.5	

α, cronbach’s alpha; k, total number of items; M, mean; SD, standard deviation

## Results

When workers were asked about their overall perception of their QOL, 2% said that they had a poor QOL, 22% said a fair QOL, 50% said a good QOL, and 26% said a very good QOL. When asked about their overall perception of their health, 2% of the workers said that they were very dissatisfied with their health, 14% were dissatisfied, 16% gave a neutral opinion, 50% were satisfied, and 18% were very satisfied. A chi-square test of independence was performed to evaluate the relationship between various demographics and alexithymia among CCU workers. No statistically significant associations were observed between alexithymia and gender, age, or marital status in the present sample, indicating that these variables were not significantly related to alexithymia prevalence. However, a significant relationship was observed between occupation and alexithymia, χ^2^ (2, *N* = 100) = 8.96, *P* = 0.011. Doctors were more likely to suffer from alexithymia compared to nurses, indicating a higher prevalence of alexithymia among medical doctors within the CCU setting (Table 2).

**Table 2:** Comparison of good and poor quality of life in association to different variables.

Variables	Good QOL (above 60)	Poor QOL (below 60)
*Physical Health*	54% (54)	46% (46)
*Psychological Health*	48% (48)	52% (52)
*Social Relations*	50% (50)	50% (50)
*Environmental health*	52% (52)	48% (48)

Additionally, the relationship between workers′ overall health status and alexithymia was significant, χ^2^ (4, *N* = 100) = 10.67, *P* = 0.031. It was observed that workers who did not report any apparent health issues had a higher frequency of alexithymia compared to those who were ill. Furthermore, specific health problems were significantly related to the probability of having alexithymia, *χ*^2^ (10, *N* = 100) = 48.08, *P* < 0.001. This suggests that certain health issues may contribute to or be associated with the likelihood of developing alexithymia. Despite these significant relationships, it was noted that alexithymia was not strongly related to physical health, as most individuals suffering from the disease did not have any type of physical health problem. However, a subset of alexithymia patients did report experiencing stress and anxiety.

The detailed data for the chi-square test are shown in Table 3.

**Table 3:** Chi-square results for alexithymia in relation to demographics.

Demographics	*f* (%)	No alexithymia	Possible alexithymia	Alexithymia present	Total	Chi-square value	*P*-Value
*Age*							
20–30	82 (82%)	36	28	18	82	4.46	0.347
31–40	16 (16%)	8	4	4	16		
41–50	2 (2%)	0	2	0	2		
Total		44	34	22	100		
*Gender*							
Male	22 (22%)	6	10	6	22	3.32	0.198
Female	78 (78%)	38	24	16	78		
Total		44	34	22	100		
*Marital status*							
Single	74 (74%)	30	30	14	74	7.71	0.103
Married	24 (24%)	12	4	4	24		
Widowed	2 (2%)	2	0	0	2		
Total		44	34	22	100		
*Occupation*							
Doctor	76 (76%)	30	24	22	76	8.96	0.011
Nurse	24 (24%)	14	10	0	24		(Significant)
Total		44	34	22	100		
*Current illness*							
Yes	30 (30%)	4	0	2	6	10.67	0.031
No	68 (68%)	40	34	18	92		(Significant)
Maybe	2 (2%)	0	0	2	2		
Total		44	34	22	100		
*Type of illness*							
Nothing	68 (68%)	36	26	6	68	48.08	<0.001
Seasonal illness (fever, flu, and gastroenteritis)	4 (4%)	2	0	2	4		(Significant)
GIT disorder (hepatitis, cholangitis, and typhoid)	8 (8%)	4	4	0	8		
Stress, anxiety, headache	6 (6%)	0	0	6	6		
Psychological issues (somatic sensory disorder and somnolence)	8 (8%)	0	4	4	8		
Rare disease (cancer, Covid, and hypothyroidism)	6 (6%)	2	0	4	6		
Total		44	34	22	100		

Table 4 shows the results of the Pearson product-moment correlation between all study variables and demographics. QOL was significantly positively correlated with overall health, particularly with physical health, psychological health, and environmental quality. These domains were positively correlated, indicating that higher scores in one domain tended to be associated with higher scores in the others, without implying causation. Additionally, QOL was positively correlated with gender, with females reporting higher scores than males.

**Table 4:** Pearson product moment correlation for study variables and demographics.

**Variables**	**1**	**2**	**3**	**4**	**5**	**6**	**7**	**8**	**9**	**10**	**11**	**12**	**13**	**14**	**15**
1. Quality of Life	–	0.65^b^	0.46^b^	0.360^a^	0.44^b^	0.45^b^	−0.22	−0.17	−0.01	−0.36	0.18	0.32^a^	0.14	0.19	0.00
2. Overall health		–	0.61^a^	0.37^b^	0.55^b^	0.49^b^	−0.36^b^	−0.32^a^	−0.28	−0.29^a^	0.15	0.41^b^	0.13	0.28	0.03
3. Physical health			–	0.71^b^	0.69^b^	0.61^b^	−0.52^b^	−0.57^b^	−0.47^b^	0.18	0.21	0.41^b^	0.17	0.28^a^	0.13
4. Social relations				–	0.57^b^	0.47^b^	−0.51^b^	−0.53^b^	−0.50	−0.17	0.46^b^	0.12	0.37^b^	0.25	0.03
5. Psychological health					–	0.62^b^	−0.48^b^	−0.42^b^	−0.36^b^	−0.38^b^	0.22	0.35^a^	0.12	0.33^a^	0.09
6. Environmental quality						–	−0.43^b^	−0.37^b^	−0.35^a^	−0.33^a^	0.14	0.44^b^	0.17	0.02	0.01
7. Alexithymia							–	0.94^b^	0.83^b^	0.63^b^	0.01	−0.12	−0.04	−0.15	0.17
8. Identifying feelings								–	0.79^b^	0.39^b^	−0.03	0.10	0.04	−0.23	0.01
9. Describing feelings									–	0.21	−0.11	0.04	−0.17	−0.24	0.11
10. Externally–oriented thinking										–	0.17	−0.15	0.12	0.16	0.25
11. Age											–	0.22	0.62^b^	0.38^b^	0.06
12. Gender												–	0.19	0.07	0.27
13. Marital status													–	0.28	−0.05
14. Occupation														–	0.08
15. Current illness															–

^a^ *P* < 0.05.

^b^ *P* < 0.01.

Overall health was significantly correlated with physical and psychological health, social relationships, and environmental quality, and was significantly negatively correlated with alexithymia and its subscales, except for difficulty in describing feelings. These correlations indicate associations between variables but do not imply directional causation. Table 4 also shows that quality of physical and psychological health was significantly better in females and nurses compared to males and doctors, respectively.

**Figure 1.**
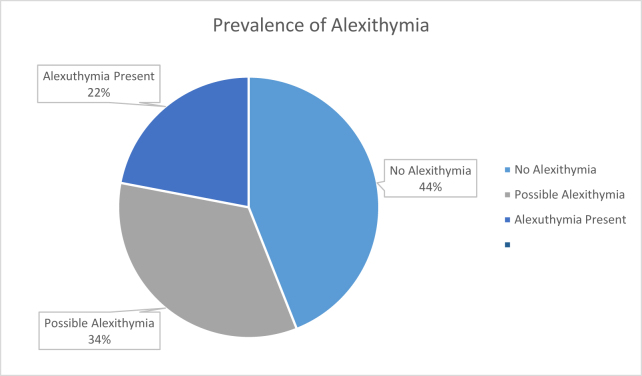
Pie graph showing the prevalence of alexithymia.

A major finding from Table 4 is that the overall QOL item was not significantly correlated with alexithymia, indicating that the QOL in both nurses and doctors remained similar regardless of the alexithymia status. However, alexithymia was significantly negatively correlated with most domain-level measures of QOL, including physical health, psychological health, and environmental quality. The social relationships domain was largely unaffected by the difficulty in describing feelings, whereas externally oriented thinking was only significantly related to psychological health and environmental quality, i.e., its presence reduced their quality but did not affect the social relationships or physical health. These results suggest that alexithymia may impair specific aspects of well-being and environmental satisfaction, even when overall life satisfaction appears unchanged. The summary of the results of the prevalence of alexithymia are illustrated in Figure 1. Lastly, alexithymia was significantly positively related to all of its subscales, i.e., difficulty in identifying and describing feelings and externally orientated thinking. This indicates that an increase in any of these factors raises the overall level of alexithymia. All of the subscales were also significantly positively correlated with each other, excluding difficulties describing feelings and externally directed thinking. This result indicates that focusing on external events and facts impaired the ability to identify one’s own feelings but did not hinder the ability to describe them. In conclusion, alexithymia significantly affects the quality of different domains of life.

## Discussion

Only few research has been done on the connection between alexithymia and the QOL of healthcare workers, particularly those who work in high-stress environments like CCUs. Our study aimed to address this significant gap by focusing on the prevalence of alexithymia in CCU personnel and its intricate implications on their QOL. Compared to other studies looking particularly at the prevalence of alexithymia in CCU nurses, our study revealed a frequency of about 22%^[^1,24^]^.

Furthermore, a more plausible reason for these differences might be the unique social and healthcare environment at different times, especially during the 2019 coronavirus disease outbreak (COVID-19). A number of psychological issues, including as alexithymia, anxiety, depression, and burnout syndrome, might arise from the increasingly demanding, stressful, and high-risk situations that ICU nurses and physicians work in^[^25^]^. Our findings regarding the relationship between alexithymia and demographic variables present both expected and surprising results.

The specific age range and career stage of CCU employees, who generally constitute a fairly homogeneous professional group in terms of educational background and job demands, may account for the lack of age-related variances in our study. In contrast to some other research, it is particularly interesting that alexithymia did not correlate with marital status, suggesting that married status was not substantially connected with emotional processing issues in this population. This disparity could be a reflection of the particular pressures that healthcare professionals encounter, when the protective benefits of marriage may be subordinated to the needs of their jobs.

The variation in alexithymia prevalence among occupational categories in the CCU context was one of our study’s most important findings. Doctors demonstrated a significantly higher likelihood of suffering from alexithymia compared to nurses. This difference may reflect variations in professional responsibilities, work-related stress, or emotional coping strategies between occupational roles; however, the current study design does not allow determination of the underlying causes. Furthermore, the substantially uneven distribution of occupational groups in our sample (more physicians than nurses) may influence prevalence estimates, and this should be considered when interpreting these results. In addition, the use of convenience non-probability sampling from hospitals within a single city may introduce sampling bias and limit the external validity of the results.

One notable finding of this study was the relationship between self-reported physical health status and alexithymia. Compared to employees who reported being physically unwell, those who did not report any obvious health problems had a greater prevalence of alexithymia. However, as health status was self-reported and those with alexithymia can have trouble identifying or properly reporting physical symptoms. Therefore, this observation should be considered exploratory and hypothesis-generating rather than a definitive pattern.

The relationship between alexithymia and QOL in our study showed a nuanced pattern. The single-item overall QOL measure was not significantly correlated with alexithymia. Although all WHOQOL-BREF domains in our study showed *significant negative associations with alexithymia*, the global item assessing overall QOL did not show a corresponding relationship. This may partly reflect psychometric characteristics of the instrument. An item response theory analysis by Vahedi (2012) demonstrated that WHOQOL-BREF items exhibit low-to-moderate discrimination and reduced measurement precision at higher levels of QOL. This suggests that certain items, particularly single-item measures, may be less sensitive in distinguishing variations among individuals with relatively preserved or higher perceived QOL. Therefore, discrepancies between domain-level impairments and the global item may arise due to limitations in measurement sensitivity rather than the absence of a clinical impact.

Although Likert-scale variables are ordinal, previous studies and simulation analyses have shown that treating 5-point or higher Likert items as continuous allows the use of parametric tests, including Pearson correlation, without substantial bias^[^26^]^. The lack of adjustment for multiple comparisons may have increased the risk of type I error, such that some statistically significant findings may represent false-positive results. Accordingly, these findings should be interpreted with caution, particularly in the context of multiple statistical tests^[^27^]^.

Higher levels of alexithymia were linked to lower scores across several dimensions of QOL, according to the strong negative associations found between alexithymia and the domains of physical health, psychological health, social interactions, and environmental quality. Theoretical concepts of alexithymia that highlight challenges in integrating emotional, cognitive, and physical experiences are generally compatible with these findings.

Additionally, quality of physical and psychological health was significantly better in females and nurses compared to males and doctors, respectively. These differences may reflect variations in emotional awareness, coping strategies, or workplace experiences between groups; however, the present study design does not allow for the determination of the underlying reasons. The pattern observed for externally oriented thinking showed that this subscale was significantly associated only with psychological health and environmental quality. This finding indicates that externally oriented thinking may be differentially related to specific domains of QOL in the present sample. However, the study design and reliance on self-reported measures limit causal interpretation and may introduce bias.

## Conclusions

The findings of this study demonstrate a notable prevalence of alexithymia among ICU healthcare workers, particularly physicians. Although alexithymia was not significantly associated with the global QOL rating, it showed significant negative correlations with several domain-specific aspects of QOL, including physical health, psychological health, social relationships, and environmental conditions. These results highlight that emotional processing difficulties may be linked with specific dimensions of well-being among ICU workers. The higher prevalence among physicians underscores the potential value of targeted interventions within medical education and practice, such as emotional intelligence training, mindfulness practices, and reflective exercises to enhance emotional awareness and expression skills. However, these findings should be interpreted cautiously due to the cross-sectional design and reliance on self-reported measures.

## Data Availability

Data are available on request from the authors.
